# Supplementary figures and images for: Patterns of Traditional and Modern Uses of Wild Edible Native Plants of Chile: Challenges and Future Perspectives
Source: Plants (Basel). 2022 Mar 11;11(6):744. doi: 10.3390/plants11060744 (PMC8953413; doi:10.3390/plants11060744)

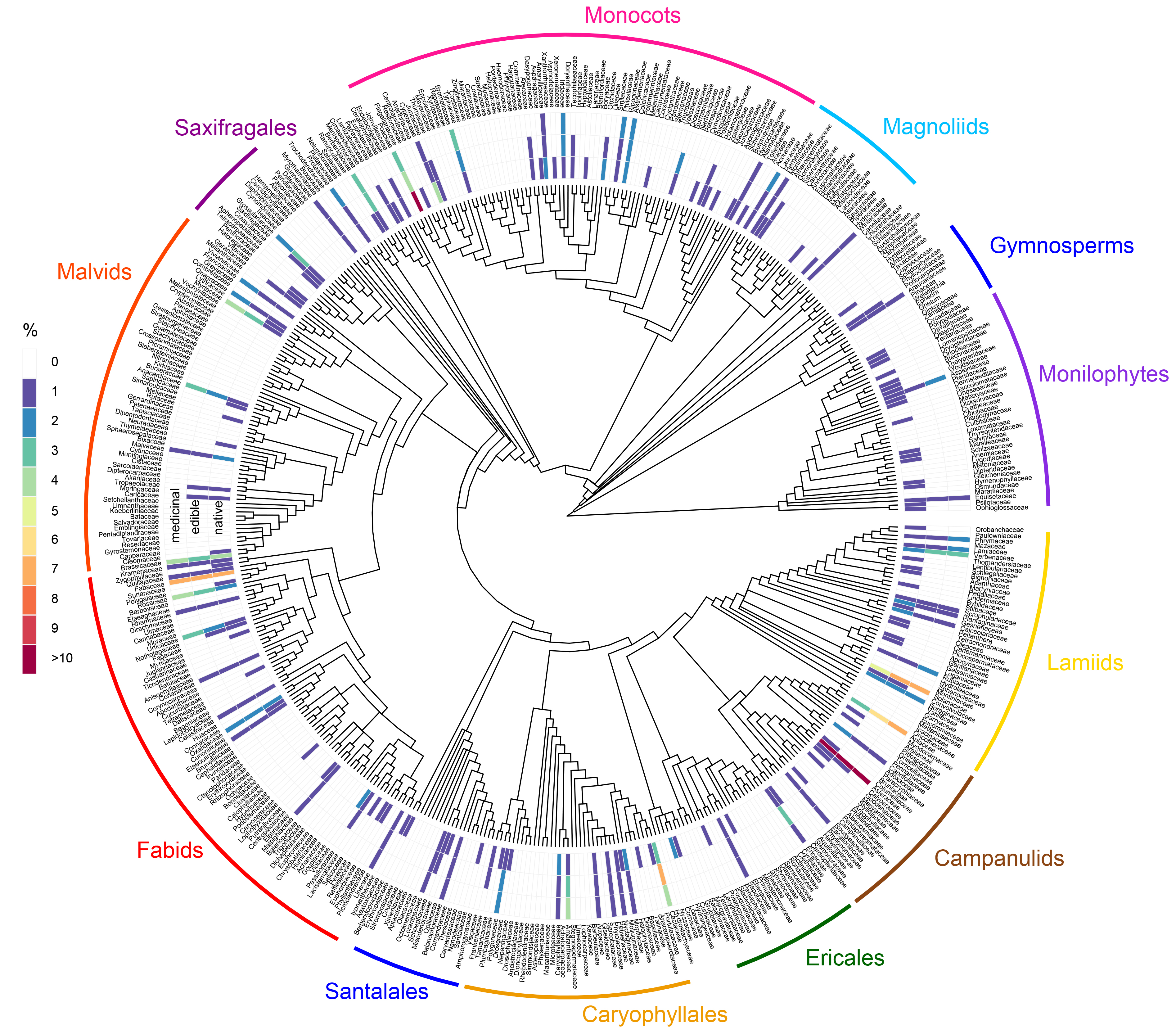

Supplement: Supplementary file 1 [file plants-11-00744-s001.zip › supplementary/Figure S1.jpg]
